# Gene Expression Analyses Implicate an Alternative Splicing Program in Regulating Contractile Gene Expression and Serum Response Factor Activity in Mice

**DOI:** 10.1371/journal.pone.0056590

**Published:** 2013-02-20

**Authors:** Twishasri Dasgupta, Samantha J. Stillwagon, Andrea N. Ladd

**Affiliations:** Department of Cellular and Molecular Medicine, Lerner Research Institute, Cleveland Clinic, Cleveland, Ohio, United States of America; INSERM, France

## Abstract

Members of the CUG-BP, Elav-like family (CELF) regulate alternative splicing in the heart. In MHC-CELFΔ transgenic mice, CELF splicing activity is inhibited postnatally in heart muscle via expression of a nuclear dominant negative CELF protein under an α-myosin heavy chain promoter. MHC-CELFΔ mice develop dilated cardiomyopathy characterized by alternative splicing defects, enlarged hearts, and severe contractile dysfunction. In this study, gene expression profiles in the hearts of wild type, high- and low-expressing lines of MHC-CELFΔ mice were compared using microarrays. Gene ontology and pathway analyses identified contraction and calcium signaling as the most affected processes. Network analysis revealed that the serum response factor (SRF) network is highly affected. Downstream targets of SRF were up-regulated in MHC-CELFΔ mice compared to the wild type, suggesting an increase in SRF activity. Although SRF levels remained unchanged, known inhibitors of SRF activity were down-regulated. Conversely, we found that these inhibitors are up-regulated and downstream SRF targets are down-regulated in the hearts of MCKCUG-BP1 mice, which mildly over-express CELF1 in heart and skeletal muscle. This suggests that changes in SRF activity are a consequence of changes in CELF-mediated regulation rather than a secondary result of compensatory pathways in heart failure. In MHC-CELFΔ males, where the phenotype is only partially penetrant, both alternative splicing changes and down-regulation of inhibitors of SRF correlate with the development of cardiomyopathy. Together, these results strongly support a role for CELF-mediated alternative splicing in the regulation of contractile gene expression, achieved in part through modulating the activity of SRF, a key cardiac transcription factor.

## Introduction

The availability of complete genome sequences and high throughput sequencing data sets has revealed that alternative splicing is an important mechanism for generating diversity from a relatively limited number of genes [1], [2]. In many cases, different protein isoforms derived by alternative splicing behave differently from one another, thereby exhibiting dissimilarities in their levels of activity or functions. Hence, tight regulation of alternative splicing is essential for appropriate temporal and spatial control of gene expression. Disruption of splicing regulation can cause or contribute to pathogenesis [3]. Dysregulated alternative splicing is associated with heart disease in mice and humans [4], [5], [6], and polymorphisms that affect alternative splicing of cardiac transcripts have been linked with susceptibility to myocardial infarction and cardiac hypertrophy [7], [8].

Members of the CUG-BP, Elav-like family (CELF) of proteins regulate alternative splicing in the heart [9]. Dysregulation of CELF-mediated alternative splicing in the heart are associated with cardiomyopathy in MHC-CELFΔ transgenic mice [10], [11]. MHC-CELFΔ transgenic mice express a nuclear dominant negative CELF protein (NLSCELFΔ) specifically in postnatal heart muscle under the control of the mouse α-myosin heavy chain promoter [10]. These mice have specific defects in CELF-mediated alternative splicing, and exhibit cardiac hypertrophy, dilated cardiomyopathy, severe cardiac dysfunction, and in some cases premature death [10], [11]. There are two lines of MHC-CELFΔ mice that express different levels of NLSCELFΔ protein: MHC-CELFΔ-10 (“severe” line) mice express higher levels and MHC-CELFΔ-574 (“mild” line) mice express lower levels [10], [11]. Both lines display dysregulation of the alternative splicing of CELF-regulated transcripts and develop cardiomyopathy, though the severe line shows a greater degree of splicing dysregulation and pathogenesis than the mild line [10], [11]. The MHC-CELFΔ phenotype can be attributed to loss of CELF activity and not exogenous protein expression, because crossing MHC-CELFΔ-10 mice with lines of MCKCUG-BP1 transgenic mice that mildly over-express CELF1 in the heart results in improved alternative splicing and reduced cardiac pathogenesis in bitransgenic offspring [10]. Studies with this model have implicated appropriate CELF-mediated alternative splicing is critical for healthy heart function, but the underlying basis of cardiomyopathy in MHC-CELFΔ mice remains unclear.

In this study, microarray analysis was performed to compare gene expression profiles in the hearts of wild type, mild and severe line MHC-CELFΔ mice. Gene ontology (GO) and pathway analyses indicated that contraction and calcium signaling were the most affected processes. Network analysis also revealed significant changes in the serum response factor (SRF) transcription factor network. Microarray, real-time RT-PCR, and western blot analyses showed a down-regulation of homeodomain only protein X (HOPX) and four and a half LIM domain-containing protein 2 (FHL2), two known inhibitors of SRF activity [12], [13], accompanied by an up-regulation of SRF targets, suggesting an increase in SRF activity. Conversely, transcript and protein levels of SRF pathway genes in a line of MCKCUG-BP1 mice that mildly over-expresses CELF1 suggest reduced SRF activity in the heart. Both alternative splicing changes and down-regulation of HOPX and FHL2 correlate with the development of overt cardiomyopathy in MHC-CELFΔ males, which exhibit partial penetrance of the phenotype. Together, these studies suggest that CELF-mediated alternative splicing is important for the regulation of contractile gene expression, and establish a role for CELF-mediated regulation in controlling the activity of SRF, a key cardiac transcription factor.

## Materials and Methods

### Ethics Statement

This study was conducted in strict accordance with the recommendations of the American Veterinary Medical Association and under the approval of the Cleveland Clinic Institutional Animal Care and Use Committee (Protocol numbers: ARC 08612 and 2011-0493). All efforts were made to minimize pain and distress during animal husbandry and euthanasia.

### Transgenic Mice

Both MHC-CELFΔ-10 and MHC-CELFΔ-574 lines of transgenic mice were maintained as hemizygotes, so wild type littermates were used for sex- and age-matched controls. Genotyping was performed by PCR as previously described [10]. Except where noted, females were used in this study because MHC-CELFΔ females exhibit higher penetrance of the phenotype than males in both lines [10]. For the comparison of unaffected and affected MHC-CELFΔ males, MHC-CELFΔ-10 males were designated “affected” if their heart size was two or more standard deviations above the mean value for age-matched wild type males as measured both by the percentage of the body weight comprised of the heart (≥0.52%) and by heart weight/tibia length ratio (≥11.15 mg/mm). MHC-CELFΔ males were designated as “unaffected” if their heart size was within one standard deviation of the mean value for wild type males for both measures (0.39–0.48% and 4.41–8.91 mg/mm).

MCKCUG-BP1 mice over-express human CELF1 in heart and skeletal muscle under the control of a muscle creatine kinase promoter [14]. Frozen heart samples had been previously collected from MCKCUG-BP1-1032 mice that were maintained as homozygotes in an FVB background. Samples from sex- and age-matched FVB/NTac wild type mice (Taconic) bred in parallel were used as controls.

### Microarray Analysis

Whole hearts were removed post-mortem from wild type and transgenic females, homogenized in Trizol (Invitrogen), and total RNA was extracted following the manufacturer’s protocol. RNA samples were DNase-treated using the DNA-free Kit (Ambion), and cleaned up using the RNeasy Kit (Qiagen). RNA concentrations and quality were confirmed on a Nanodrop ND-1000 spectrophotometer and Agilent Bio-analyzer. cDNA library preparation and hybridization to MouseWG-6 v2.0 Expression BeadChips containing >45,000 probes based on RefSeq release 22 and supplemented with MEEBO and RIKEN FANTOM2 content (Illumina) were performed by the Cleveland Clinic Lerner Research Institute’s Genomics Core. The microarray data for this study have been deposited in NCBI’s Gene Expression Omnibus [15], and are accessible through GEO Series accession number GSE40677 (http://www.ncbi.nlm.nih.gov/geo/query/acc.cgi?acc=GSE40677).

Statistical comparisons of the data, pathway and gene ontology analyses were performed by the Cleveland Clinic Bioinformatics Core. The summarized data from the raw microarray data were log2 transformed and processed with background correction and quantile normalization. Quality control analyses were applied to detect the outlier samples. Linear models and empirical Bayes method in Limma [16] were used to access the differential expression between the wild types and transgenics. Those genes that satisfied the FDR p-value threshold of <0.05 or raw p-value of <0.001 and absolute fold change threshold of 2.0 were identified as significant for the functional pathway and network analyses. Functional profiling of differentially affected biological processes, functions, and pathways between the transgenic and control samples were evaluated using both publicly available tools (NCBI, GOTermFinder, GOTermMapper, MGI, and KEGG) and the commercial pathway analysis databases Metacore (Metacore™) and Ingenuity Pathway Analysis (Ingenuity® Systems).

To identify significantly enriched gene ontology (GO) terms among the genes of interest, the web-based Gene Ontology Enrichment Analysis Software Toolkit (http://omicslab.genetics.ac.cn/GOEAST/) was used to compare the list of significantly affected genes against the Illumina Refseq 8 background gene set [17]. Graphical outputs from GO analyses are structured as directed acyclic graphs [17]. Boxes represent GO terms, and are labeled with GO ID, term definition, and P value. Arrows represent relationships between more and less specialized terms. Branches of the GO hierarchical tree without significantly enriched GO terms are not shown. The colors of the boxes reflect enrichment in the mild, severe, or both mild and severe lines. The degree of color saturation of each node positively correlates with the significance of enrichment of the corresponding GO term. The degrees of saturation of boxes representing common GO terms are determined by the lower of the P values for those terms. Separate analyses were performed for biological process and cellular component categories.

### Real-time RT-PCR

Two µg of total RNA was reverse transcribed in a 20 µl reaction volume using Superscript-VILO cDNA Synthesis Kit (Invitrogen). PCR reactions contained 2.5 pmol of forward and reverse primers (Table S1), 12 µl of Power SYBR Green Master Mix (Applied Biosystems), and 5 ng of cDNA brought up to 25 µl with nuclease-free water. Glyceraldehyde 3-phosphate dehydrogenase (GAPDH) was used as a control, as GAPDH transcript levels showed no differences between wild type and transgenic samples on microarrays. All real-time RT-PCR reactions were performed in triplicate using a StepOne Plus instrument (Applied Biosystems). PCR was performed at 95°C for 10 min, followed by 40 cycles at 95°C for 15 sec and 60°C for one min. A DNA melting curve was generated after the PCR cycles in order to discriminate between specific amplicon and non-specific amplification products. Data was analyzed with StepOne software v2.1 using the comparative CT method. Fold changes are reported as mean+standard error of the mean. Statistical comparisons of means were performed via one-tailed t-tests assuming unequal variances using Microsoft Excel software. Differences were considered statistically significant when P ≤ 0.05.

### Western Blotting

Whole hearts were homogenized in protein loading buffer (0.64 M Tris-HCl [pH 6.8], 10% glycerol, 2% sodium dodecyl sulphate, and 5% β-mercaptoethanol), and total protein was quantified using the Non-interfering Protein Assay Kit (G-Biosciences). The samples were resolved by SDS-polyacrylamide gel electrophoresis and transferred to Immobilon-P membranes (Millipore). Membranes were probed as previously described [18] and visualized with Immobilon Western Chemiluminescent HRP Substrate (Millipore). Primary and secondary antibodies used are listed in Table S2. Equivalent loading was confirmed both by Ponceau S staining and GAPDH expression.

### Alternative Splicing RT-PCR

Total RNA was harvested from wild type and transgenic hearts and alternative splicing was analyzed by single-tube RT-PCR as previously described [10]. Primers and reaction conditions were previously published for *Mef2A* exon 16 and *Itgb1* exon D [10] and *Ank2* exon 21 [19].

## Results

### Repression of CELF-mediated Alternative Splicing Leads to Specific Changes in Gene Expression

To determine how gene expression is affected at a global level in MHC-CELFΔ mice, we performed microarray analysis on total RNA extracted from whole hearts of three MHC-CELFΔ-10 and three MHC-CELFΔ-574 females collected at weaning (three weeks old). Three female wild type littermates from each line were used as controls. The three-week time point was chosen to minimize the contributions of secondary effects from compensation/decompensation that may occur at later stages of pathogenesis. Around birth, expression of the dominant negative protein and changes in alternative splicing are not detected, and the hearts of MHC-CELFΔ mice are indistinguishable from their wild type littermates by histology or functional tests [10], [11]. Three weeks after birth, however, expression of the transgene and alternative splicing defects are evident. Cardiac dysfunction is maximal in both lines, but pathological changes in the gross morphology and histology seen in the severe line (e.g., fibrosis) are less overt than at later stages [10], [11]. Hierarchical clustering using correlation distances in raw, normalized, and quality control-filtered data sets showed consistent clustering of wild type samples from both lines together (Figure S1), so all wild type samples were pooled (total n = 6) for subsequent analyses. Wild type versus mild line and wild type versus severe line comparisons were made, and differentially expressed genes that exhibit an absolute fold change ≥2 were identified.

**Figure 1:**
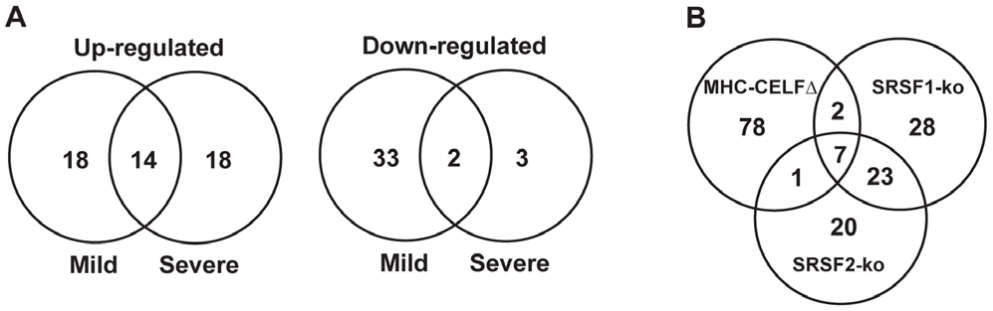
Venn diagram comparisons of affected genes in MHC-CELFΔ hearts. (A) Respective overlap of affected up-regulated and down-regulated genes between mild and severe MHC-CELFΔ lines. (B) Affected genes in MHC-CELFΔ hearts were compared to affected genes in two cardiac-specific knockout models (SRSF1-ko and SRSF2-ko) lacking one of the SR proteins, SRSF1 or SRSF2 (also known as ASF/SF2 and SC35, respectively).

The results of these comparisons showed that the majority of genes are expressed relatively normally in MHC-CELFΔ hearts. In total, 88 genes were identified that were up- or down-regulated (50 and 38 genes, respectively) at least two-fold in MHC-CELFΔ hearts compared with wild type (Table S3). Of these, 67 were affected in the mild line and 37 in the severe line, with an overlap of 16 genes (Figure 1A). Of the genes uniquely identified in the mild line, over half (12 out of 18 up-regulated genes and 18 out of 33 down-regulated genes) were similarly affected in the severe line, but with a fold change that fell below the two-fold cut-off (Table S3). Likewise, many of the genes identified only in the severe line (6 out of 18 up-regulated genes and 2 out of 3 down-regulated genes) displayed similar but less than two-fold changes in the mild line. Only one gene, *Erdr1*, showed opposing regulation between the two lines, being down-regulated more than two-fold in the severe line, but up-regulated slightly (∼1.5-fold) in the mild line.

Microarray analyses of heart tissues have also been published from two cardiac-specific splicing factor knockout mouse models, each lacking one member of the serine/arginine-rich (SR) family in the heart, SR splicing factor 1 (SRSF1, formerly known as ASF/SF2) or SRSF2 (formerly known as SC35) [20], [21]. Both models develop dilated cardiomyopathy [20], [21]. Of 60 genes significantly altered in SRSF1-knockout mice and 51 genes significantly altered in SRSF2-knockout mice, 30 genes were similarly affected in both (28 up-regulated and 2 down-regulated). This high degree of overlap led Xu and colleagues to propose that many of the changes observed in the SR protein-deficient hearts were part of a common compensatory pathway associated with dilated cardiomyopathy [21]. As MHC-CELFΔ mice also develop dilated cardiomyopathy, we compared the changes observed in MHC-CELFΔ hearts to those in the SR protein knockout models (Table S4). Strikingly, of the genes that were similarly dysregulated in both SRSF1- and SRSF2-knockout mice, more than three quarters (23 out of 30) were not significantly affected in either line of MHC-CELFΔ mice. Conversely, of the 88 genes significantly affected in one or both MHC-CELFΔ lines, only seven (*Acta1*, *Ctgf*, *Col8a1*, *Fhl1*, *Timp1*, *Nppb*, and *Ces3*) were similarly affected in both SR protein-deficient models, with another three (*Tnnt1*, *Gdf15*, and *Ryr2*) affected in either SRSF1- or SRSF2-knockout mice (Figure 1B). Nearly 90% (78 out of 88) of the genes affected in MHC-CELFΔ hearts were not significantly affected in either of the SR protein knockout models. The small degree of overlap with the SR protein-knockout models suggests that most of the changes observed in the MHC-CELFΔ mice were not due to shared pathways of dilated cardiomyopathy or heart failure.

### Expression of Genes Involved in Contraction and Contractility is Altered in the Hearts of MHC-CELFΔ Mice

**Figure 2:**
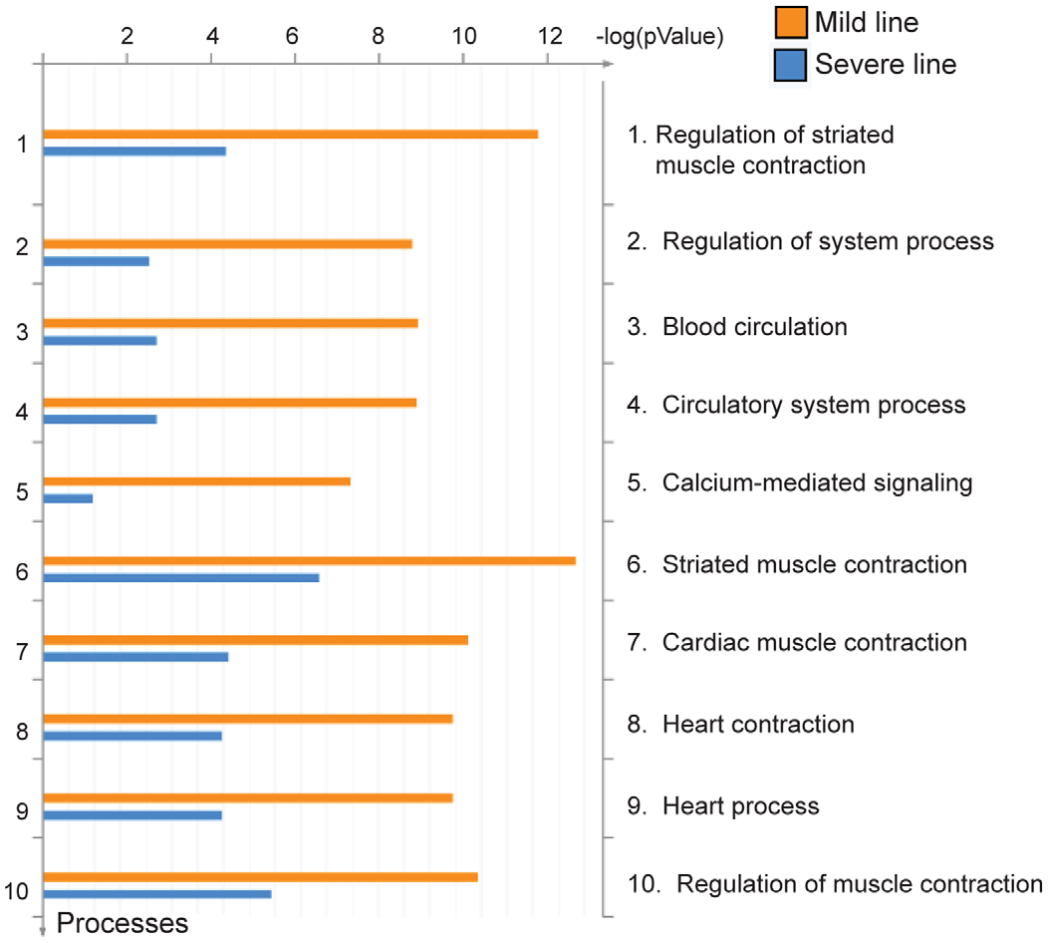
Functionally enriched processes identified in MHC-CELFΔ hearts. The top ten most functionally enriched processes in MHC-CELFΔ versus wild type hearts identified using the Metacore™ pathway analysis database are shown.

Functional profiling was performed on the 88 genes significantly affected in the hearts of one or both lines of MHC-CELFΔ mice to identify processes and pathways differentially affected by dysregulation of CELF-mediated alternative splicing. Five of the top ten functionally enriched processes identified using the Metacore pathway analysis database relate to muscle contraction or regulation of muscle contraction (Figure 2). Calcium-mediated signaling was also highly affected, which is significant since it is the release and uptake of calcium from intracellular stores that links excitation with contraction and relaxation in striated muscle. Calcium signaling was also the top canonical pathway identified using Ingenuity Pathway Analysis software for both the mild and severe line (P = 1.98E−06 for the mild line versus wild type, and P = 1.51E−04 for the severe line versus wild type).

Gene ontology (GO) enrichment analysis was also performed to determine the most affected biological processes and cellular components. Genes related to the biological processes of muscle differentiation, myofibril assembly, calcium ion homeostasis, and calcium-mediated signaling were enriched in both lines (Figure S2). Genes involved in cation transport, regulation of blood pressure, and Wnt signaling were also enriched in the mild line, whereas genes involved in glucose catabolism were differentially enriched in the severe line. Cellular components of the actin cytoskeleton, myofibril, and extracellular matrix were enriched in both lines (Figure S3). Genes related to the sarcoplasmic reticulum, which is the primary intracellular calcium storage site in striated muscle, were also enriched in the mild line. Collagen matrix genes were enriched in the severe but not mild line, consistent with the presence of fibrosis only in this line [10], [11]. Together, these analyses all point to a direct disruption in the expression of contraction, contractility, and calcium handling genes in the MHC-CELFΔ heart. This is consistent with the MHC-CELFΔ phenotype, in which Doppler ultrasound analysis and echocardiography revealed impaired contractile function [10], [11]. Notably, gene expression changes that would support other possible causes of poor cardiac function, such as mitochondrial failure or severe metabolic defects, were not observed.

### The Serum Response Factor Pathway is Affected by the Loss of CELF Activity

**Figure 3:**
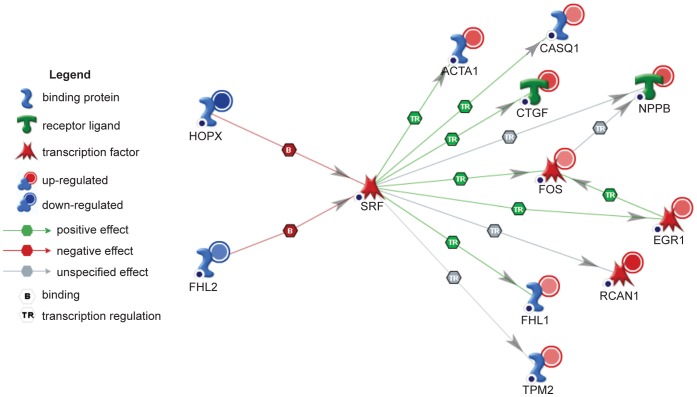
The serum response factor (SRF) network is affected in MHC-CELFΔ mice. The SRF network was identified as the most affected transcription factor network in MHC-CELFΔ mice using Ingenuity Pathway Analysis software. Two up-stream inhibitors of SRF activity, *Hopx* and *Fhl2*, are down-regulated (blue circles) in MHC-CELFΔ hearts, while nine down-stream targets of SRF are up-regulated (red circles). *Srf* levels are unchanged. The saturation level (blue and red circles) reflects the relative fold change in MHC-CELFΔ mice relative to wild type.

In addition to the pathway and gene ontology analyses, network analysis was performed using Ingenuity Pathway Analysis software. The SRF network was the most affected transcription factor network in MHC-CELFΔ mice (P = 7.75E−35). In this network, nine known targets of SRF regulation (*Acta1*, *Casq1*, *Ctgf*, *Nppb*, *Fos*, *Egr1*, *Rcan1*, *Fhl1*, and *Tpm2*) were up-regulated in MHC-CELFΔ mice (Figure 3 and Table 1), suggesting an increase in SRF activity. The levels of *Srf*, however, did not change. *Hopx* and *Fhl2*, genes that encode proteins known to bind to SRF and inhibit its activity [12], [22], [23], were down-regulated.

**Table 1:** Microarray results for genes in the serum response factor (SRF) pathway in MHC-CELFΔ hearts^a^.

		MILD LINE					SEVERE LINE				
Gene	Protein AKA	logFC	APV	DOC	FC from WT	|FC|	logFC	APV	DOC	FC from WT	|FC|
*Srf*		0.20	0.3155	None	1.15	1.15	0.14	0.5113	None	1.10	1.10
*Acta1*		1.42	2.59E−08	Up	2.68	2.68	1.17	3.06E−07	Up	2.26	2.26
*Casq1*		1.10	0.0011	Up	2.14	2.14	0.59	0.0818	Slight up (n.s.)	1.51	1.51
*Ctgf*		1.35	0.0009	Up	2.55	2.55	1.54	0.0004	Up	2.91	2.91
*Nppb*	BNP	1.44	0.0014	Up	2.71	2.71	0.88	0.0486	Slight up	1.84	1.84
*Fos*	c-fos	1.01	0.0108	Up	2.01	2.01	0.27	0.6673	Slight up (n.s.)	1.20	1.20
*Egr1*		1.28	0.0004	Up	2.44	2.44	0.49	0.2038	Slight up (n.s.)	1.40	1.40
*Rcan1*	calcipressin	1.89	5.59E−06	Up	3.69	3.69	2.15	2.03E−06	Up	4.43	4.43
*Fhl1*	SLIM1	0.46	0.0189	Slight up	1.37	1.37	1.02	3.10E−05	Up	2.02	2.02
*Tpm2*		0.53	0.0026	Slight up	1.45	1.45	1.12	3.64E−06	Up	2.18	2.18
*Hopx*	LAGY	−3.00	8.63E−12	Down	0.13	8.00	−0.51	0.0025	Slight down	0.70	1.43
*Fhl2*	SLIM3	−1.66	1.57E−10	Down	0.32	3.17	−0.70	7.64E−06	Slight down	0.62	1.62

a AKA = also known as, FC = fold change, APV = adjusted P value, DOC = direction of change, WT = wild type, |FC| = absolute fold change, n.s. = not significant.

**Figure 4:**
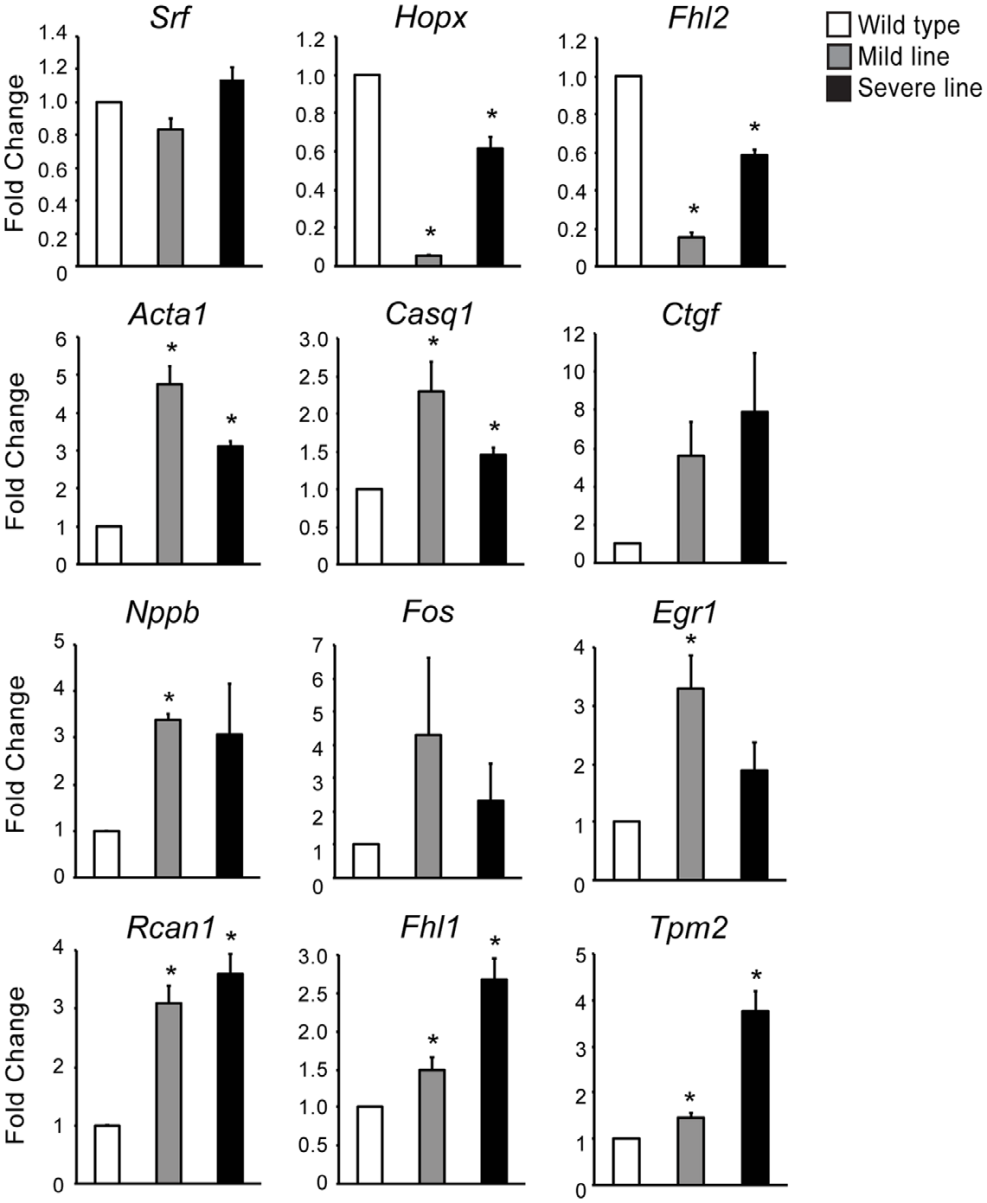
Real-time RT-PCR validation of the microarray results for the SRF network genes. Total RNA was extracted and mRNA levels were assayed using SYBR green-based detection. Reduced levels of upstream inhibitors of SRF and elevated levels of downstream targets of SRF were confirmed in MHC-CELFΔ hearts compared to wild type, while *Srf* transcript levels remain unchanged. Fold changes shown represent the mean+standard error of the mean of three independent sample sets tested in independent experiments. Similar directional changes or lack thereof were observed for each transcript in all three sample sets. An asterisk indicates the mean is significantly different from that of the wild type (P ≤ 0.05).

To validate the microarray results, real-time RT-PCR analysis was performed on all twelve SRF network genes in wild type and MHC-CELFΔ heart samples (Figure 4). The transcript levels of both the upstream inhibitors, *Hopx* and *Fhl2*, were significantly decreased in the mild and severe MHC-CELFΔ lines compared to the wild type, with fold changes that were similar to the microarray results (Table 1). *Srf* transcript levels remained unchanged between wild type and the two transgenic lines. Since HOPX and FHL2 are known to repress SRF activity, the reduced levels of these two factors would be predicted to increase the activity of SRF, leading to the up-regulation of its downstream targets. An increase in the transcript levels of the nine downstream SRF targets identified in this network (*Acta1*, *Casq1*, *Ctgf*, *Nppb*, *Fos*, *Egr1*, *Rcan1*, *Fhl1*, and *Tpm2*) was confirmed, supporting an increase in SRF activity. Although the increases in mean *Ctgf* and *Fos* transcript levels did not reach statistical significance (P = 0.06 and 0.07 for *Ctgf* in the mild and severe line, respectively, and P = 0.14 and 0.18 for *Fos*), this is likely due to higher levels of variability for these transcripts. Some degree of increase was observed for both transcripts in all three of the three sample sets tested, ranging from a 2.1- to 8.1-fold increase for *Ctgf* and from 1.4- to 8.9-fold for *Fos*.

**Figure 5:**
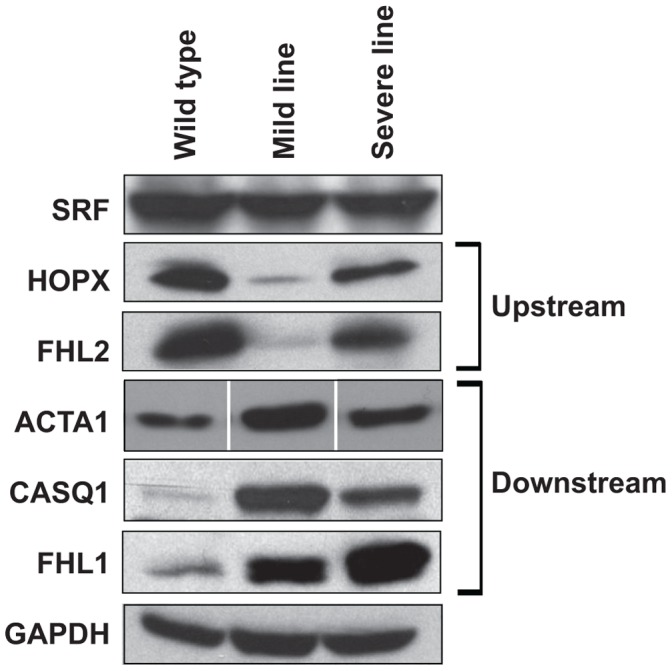
Protein expression of SRF network genes in wild type, mild and severe MHC-CELFΔ lines. Western blot analysis shows a decrease in expression of the SRF inhibitors HOPX and FHL2 in the mild and severe lines of MHC-CELFΔ mice compared to wild type, accompanied by an increase in the levels of three SRF targets. SRF and GAPDH expression levels are constant. Equivalent loading was further confirmed by Ponceau S staining (data not shown). Representatives of three independent blots with similar results are shown. Irrelevant intervening lanes were excised on the ACTA1 blot.

Western blot analyses were also performed on SRF, HOPX, FHL2, and three of the downstream targets of SRF: ACTA1, CASQ1, and FHL1 (Figure 5). Decreases in HOPX and FHL2 protein expression were observed in both the mild and severe MHC-CELFΔ lines compared to the wild type. In contrast, the protein levels of the downstream SRF targets were increased in the transgenic lines relative to wild type. SRF expression levels were the same. These data indicate that the changes in transcript levels in the SRF network reflect comparable changes in protein expression.

### CELF1 Over-expression Up-regulates Fhl2, Leading to an Apparent Decrease in SRF Activity

**Figure 6:**
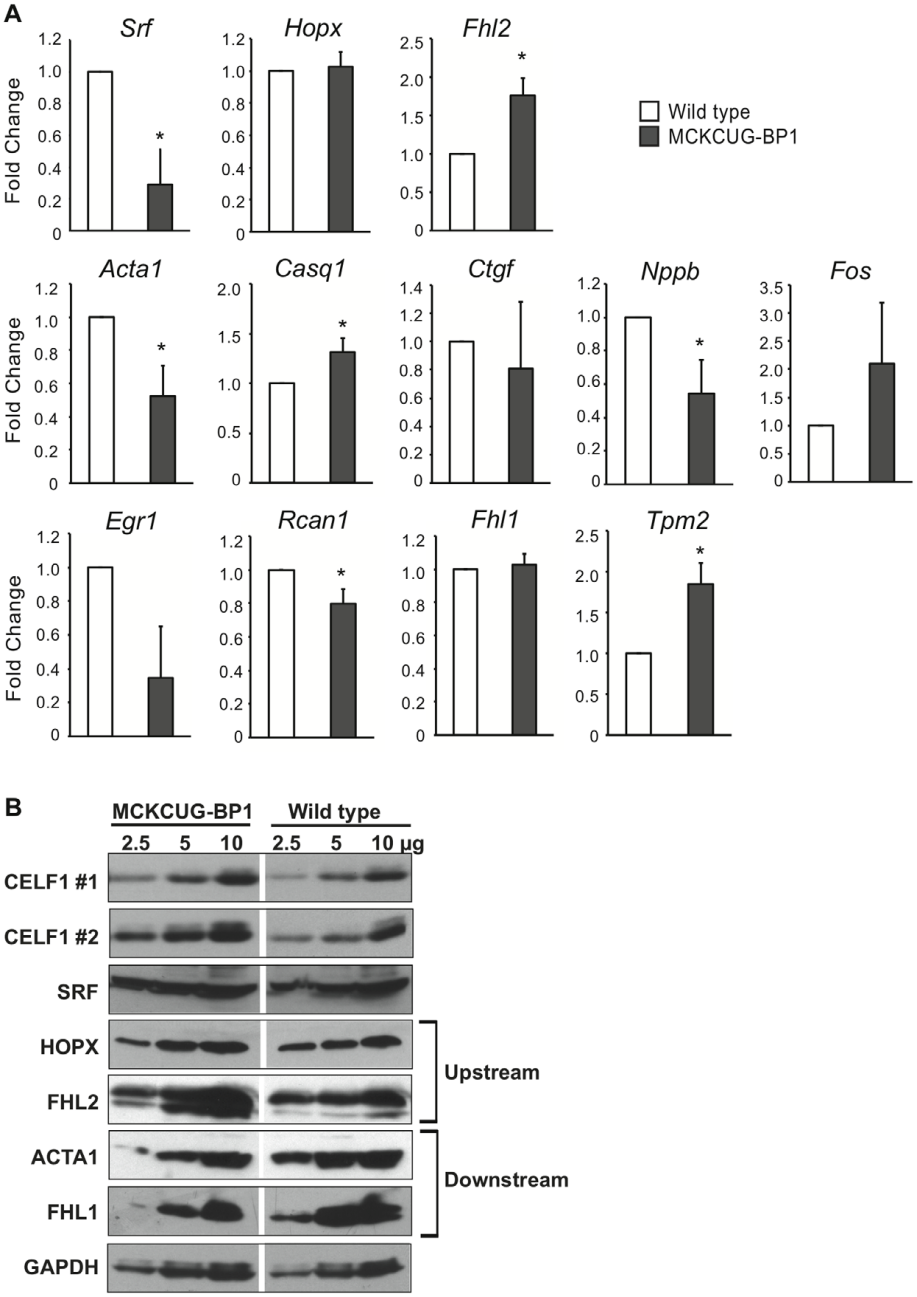
Expression of SRF network genes in wild type and MCKCUG-BP1 hearts. (A) Total RNA was extracted from adult wild type and MCKCUG-BP1 hearts and mRNA levels were assayed using SYBR green-based detection. A general trend shows an increase in the mRNA levels of *Fhl2*, the upstream inhibitor of SRF, and a decrease in the levels of some of the downstream targets of SRF in MCKCUG-BP1 hearts compared to wild type, an effect opposite to that seen in MHC-CELFΔ mice. *Srf* transcripts are also decreased in MCKCUG-BP1 hearts relative to wild type. Fold changes shown represent the mean+standard error of the mean of four independent sample sets tested in independent experiments. An asterisk indicates the mean is significantly different from that of wild type (P ≤ 0.05). (B) Dilutions of total protein from adult MCKCUG-BP1 and wild type hearts were probed on the same blot. Empty or irrelevant lanes between the MCKCUG-BP1 and wild type samples were excised. The level of CELF1 over-expression varied from ∼1.5 fold (CELF1 #1) to ∼2–3 fold (CELF1 #2). An increase in the protein level of the upstream inhibitor of SRF, FHL2, and a decrease in the levels of two downstream SRF targets establish a general pattern opposite to that observed in MHC-CELFΔ hearts. Representatives of three independent sets are shown; similar differences were observed in two out of three MCKCUG-BP1 samples relative to wild type, and correlated with higher levels of CELF1 over-expression. SRF and GAPDH protein levels are unchanged in all samples tested. Equivalent loading was further confirmed on each blot by Ponceau S staining (data not shown).

To establish that the cause for the activation of the SRF transcription factor pathway is the change in CELF activity in MHC-CELFΔ mice and not secondary to the development of cardiomyopathy and heart failure, the consequences of CELF1 over-expression on the SRF pathway was examined in heart samples from a line of MCKCUG-BP1 mice that mildly over-expresses CELF1 in heart muscle, but does not develop overt cardiomyopathy [14]. Similar to previous experiments with MHC-CELFΔ heart samples, real-time RT-PCR analysis was performed for all twelve SRF network genes to compare their transcript levels in sex- and age-matched wild type and MCKCUG-BP1 heart samples (Figure 6A). Western blots were also performed for CELF1, SRF, HOPX, FHL2, ACTA1, CASQ1, FHL1, and GAPDH (Figure 6B).

The MCKCUG-BP1-1032 line used in this study has been reported to express a total (i.e., endogenous plus exogenous) of ∼1.5 times the normal level of CELF1 protein in the heart [14]. This is not enough to cause an overt cardiac phenotype [14], but it is sufficient over-expression to partially rescue cardiac pathogenesis in MHC-CELFΔ-10 mice [10]. The over-expression of CELF1 in the MCKCUG-BP1 heart was confirmed by western blot. Notably, we observed some variability in the level of over-expression, ranging from ∼1.5-fold as previously reported to ∼2- to 3-fold (Figure 6B).

We postulated that increased CELF1 expression would boost *Hopx* and *Fhl2* levels, thereby reducing SRF activity without changing the levels of SRF. Surprisingly, a decrease in *Srf* transcript levels was seen with CELF1 over-expression in three out of four MCKCUG-BP1 samples (Figure 6A). Western blot analysis, however, did not show a corresponding difference in SRF protein levels between wild type and MCKCUG-BP1 mice in any individuals tested (Figure 6B). The transcript levels of *Fhl2* were increased in MCKCUG-BP1 lines compared to the wild type, but *Hopx* transcript levels were unchanged (Figure 6A). Likewise HOPX protein levels did not noticeably differ between MCKCUG-BP1 and wild type hearts, whereas FHL2 protein levels were elevated in two individuals that had approximately 2-to 3-fold over-expression of CELF1, but did not change in a third that had only ∼1.5-fold over-expression (Figure 6B and data not shown).

Transcript levels of the downstream SRF targets *Acta1*, *Nppb*, and *Rcan1* were reduced in MCKCUG-BP1 hearts (Figure 6A). Two additional SRF targets (*Ctgf* and *Egr1*) showed a trend towards decreased expression in the MCKCUG-BP1 heart, but did not reach statistical significance. For both *Ctgf* and *Egr1*, expression of the target was reduced relative to wild type levels in three out of four MCKCUG-BP1 samples, though to a variable degree. Concordant with the reduced transcript levels, ACTA1 protein levels decreased in two out of three MCKCUG-BP1 samples, correlating with the level of over-expression of CELF1 (Figure 6B and data not shown). *Fhl1* transcript levels did not exhibit consistent down-regulation in the MCKCUG-BP1 heart RNA samples tested (Figure 6A), but FHL1 protein levels were somewhat reduced in the same two out of three MCKCUG-BP1 individuals that have higher levels of CELF1 and reduced levels of ACTA1 (Figure 6B and data not shown). Notably, five of the targets that are down-regulated in MCKCUG-BP1 mice (*Acta1*, *Nppb*, *Egr1*, *Rcan1*, and *Fhl1*) were reduced in primary murine cardiomyocytes following ablation of SRF [24]. In addition, two SRF targets (*Casq1* and *Tpm2*) had higher transcript levels (Figure 6A), with three out of four MCKCUG-BP1 samples showing variable levels of up-regulation. *Fos* also exhibited a trend towards increased expression, but was only elevated in two out of four samples. Although *Casq1* transcript levels appear to be slightly elevated in the MCKCUG-BP1 heart, no effect was seen on CASQ1 protein levels in any of the samples regardless of the level of CELF1 over-expression (data not shown). Overall, seven of the eleven SRF network genes dysregulated in response to repression of CELF activity (*Fhl2, Acta1*, *Ctgf*, *Nppb*, *Egr1*, *Rcan1*, and *Fhl1*) showed a reverse trend in CELF1 over-expressing hearts. Furthermore, these changes appear to be dose-dependent (i.e., greater over-expression of CELF1 correlates with greater changes in transcript and protein expression).

### Changes in CELF-mediated Alternative Splicing and SRF Inhibitor Expression Correlate with Cardiomyopathy in MHC-CELFΔ Males

**Figure 7:**
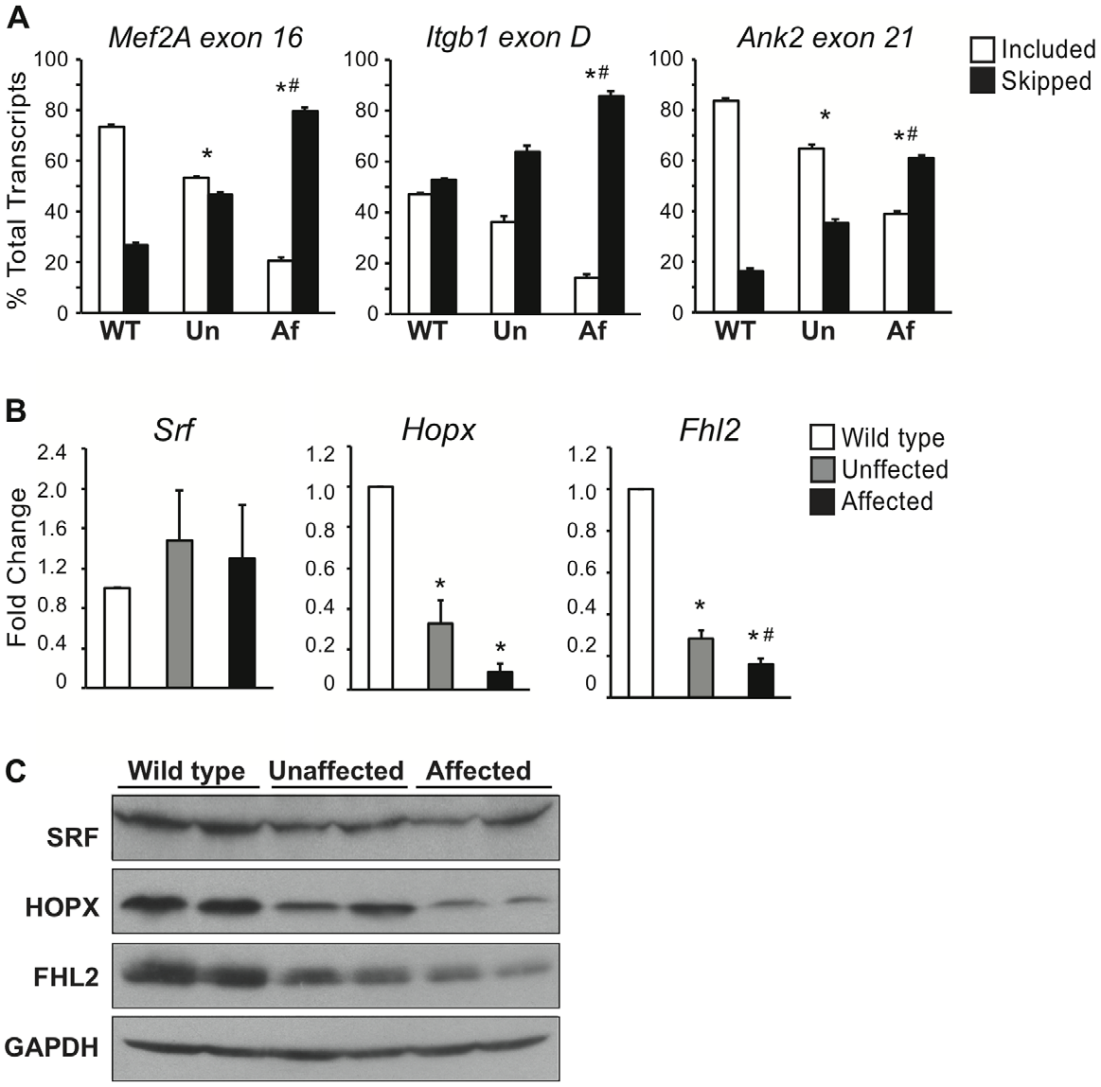
Dysregulation of alternative splicing and SRF inhibitor expression correlates with cardiomyopathy in MHC-CELFΔ males. (A) Total RNA was harvested from the hearts of adult wild type (WT), unaffected (Un) and affected (Af) MHC-CELFΔ severe line males. The alternative splicing of *Mef2A* exon 16, *Itgb1* exon D, and *Ank2* exon 21 were evaluated by RT-PCR. (B) *Srf*, *Hopx*, and *Fhl2* transcript levels were assessed in unaffected and affected MHC-CELFΔ severe line males by real time RT-PCR. For (A) and (B), mean values+standard error of the mean are shown for three individuals per group. An asterisk indicates the mean is significantly different from that of wild type, whereas a pound sign indicates a significant difference between affected and unaffected males (P ≤ 0.05). Both changes in alternative splicing and transcript levels show a graded response that correlates with the incidence of cardiomyopathy in MHC-CELFΔ males. (C) Total protein was harvested from wild type, unaffected, and affected MHC-CELFΔ severe line males and SRF, HOPX, FHL2, and GAPDH levels were assessed by western blot. HOPX and FHL2 levels are down-regulated to a greater extent in affected than unaffected males, whereas SRF protein levels were similar in unaffected and affected males. Equivalent loading is indicated by constant levels of GAPDH, and was further confirmed by Ponceau S staining (data not shown). Representative blots showing samples from two out of four individuals tested from each group are shown.

To determine whether the dysregulation of alternative splicing and/or SRF correlates with the degree of pathogenesis, we examined both alternative splicing of known CELF targets and levels of SRF, HOPX, and FHL2 in the hearts of adult MHC-CELFΔ severe line males. While all severe line MHC-CELFΔ females exhibit changes in CELF-mediated alternative splicing and develop cardiomyopathy by 24 weeks of age, only about half of severe line transgenic males do so [10]. Suggestively, it was previously shown that the fraction of severe line males with altered *Mtmr1* alternative splicing is similar to the fraction that develop enlarged hearts [10], but in these experiments alternative splicing was assessed independently from pathogenic changes. Here, 24 week-old males were designated “affected” if their heart size was two or more standard deviations above the mean value for age-matched wild type males, and “unaffected” if their heart size was within one standard deviation of normal. The alternative splicing of three known CELF targets, *Mef2A* exon 16, *Itgb1* exon D, and *Ank2* exon 21, were strongly dysregulated in affected males (Figure 7A), similar to age-matched severe line females ([11] and Figure S4). Strikingly, the alternative splicing patterns of these targets were significantly less disrupted in unaffected males (Figure 7A).

Transcript levels of *Hopx* and *Fhl2* were lower in MHC-CELFΔ severe line males than wild type, but were reduced more in affected than unaffected transgenic males (Figure 7B). *Srf* transcript levels did not differ from wild type. Consistent with these results, HOPX and FHL2 proteins both exhibited a graded down-regulation in transgenic males, with affected males having lower levels than unaffected males (Figure 7C). SRF protein levels did not differ between unaffected and affected males, though a slight reduction compared to wild type was observed.

## Discussion

The present gene expression profiling study implicates dysregulation of contractile gene expression programs as a primary cause of cardiomyopathy and cardiac dysfunction in MHC-CELFΔ mice. The dominant negative protein expressed in the hearts of these mice is restricted to the nucleus [10]. Thus, dysregulation of CELF-mediated alternative splicing likely underlies these changes, although disruption of other nuclear functions of the CELF proteins (e.g., RNA editing [9]) cannot be ruled out. Although this type of microarray does not reveal changes in alternative splicing, a few CELF targets identified to date encode proteins with known roles in contraction. For example, ankyrin 2 (ANK2) is required for targeting and stability of the Na^+^/Ca^2+^ exchanger 1 in cardiomyocytes [25]. Loss-of-function has been implicated in inherited cardiac arrhythmia with increased risk for sudden cardiac death, likely due to elevation of calcium transients [26], [27], [28]. Alternative splicing of *Ank2* exon 21 was previously found to be regulated during heart development, and to respond to CELF1 over-expression [19]. *Ank2* alternative splicing is altered in the MHC-CELFΔ severe line, but not the mild line (Figure S4).

Another striking finding of our current study is the dysregulation of the SRF transcription factor network in CELF-repressed and over-expressing mice. SRF is recognized for being at the convergence of multiple signaling pathways, known not only for controlling the transcription of immediate early response genes, such as *Fos* and *Egr1* [29], but also for the transcriptional activation of a large number of cardiac genes involved in contractile apparatus structure and function [24]. Regulation by SRF is a common feature of many genes up-regulated during cardiac hypertrophy [30]. Heart-specific over-expression of SRF in transgenic mice leads to the development of cardiac hypertrophy, chamber dilation, and impaired cardiac function [31], not very dissimilar from what we observe in MHC-CELFΔ mice. SRF levels normally increase slightly in the heart with age, and low levels of over-expression in the heart mimic cardiac aging, including myocardial cell hypertrophy and reduced cardiac function [32]. Over-expression of SRF alone activates only a subset of its targets, however, and is not sufficient to induce the full hypertrophic gene expression profile in cardiomyocytes [30]. SRF has low inherent transactivation activity by itself, but has strong transcriptional activity when working in concert with other transcription factors [33]. Several other transcription factors that have been implicated in cardiac hypertrophy, including Nkx2.5 and HAND proteins [34], were not identified in the network analysis of our microarray data. A regulatory network for GATA4 was identified as dysregulated in the MHC-CELFΔ heart, but the handful of GATA4 targets affected exhibited a mixture of up- and down-regulation, together failing to suggest a consistent increase or decrease in GATA4 activity (data not shown).

CELF-mediated changes in SRF activity can likely be attributed to the levels of the inhibitory proteins, HOPX and FHL2, as the levels of SRF protein and alternative splicing of *Srf* transcripts did not differ between wild type and transgenic animals (Figures 5 and 6, and data not shown). Over-expression of CELF1 did lead to a reduction in *Srf* transcript levels, but SRF protein levels were maintained, suggesting there may be a compensatory mechanism in place to maintain SRF levels in the myocardium. HOPX negatively regulates SRF activity by directly interacting with the SRF protein and inhibiting its binding to DNA [22], [23]. Ablation of HOPX is sufficient to induce an up-regulation of some SRF target genes, and can lead to cardiac hypertrophy in mice [23]. FHL2 likewise inhibits transactivation of transcription by SRF via a direct protein:protein interaction [12]. FHL2 knockout mice undergo normal cardiac development, but exhibit an exaggerated hypertrophic response following β-adrenergic stimulation [35]. Both *HOPX* and *FHL2* are down-regulated in human heart failure [36], [37], and may contribute to the dysregulation of SRF-dependent gene expression during pathogenesis. Not all of the SRF targets that were up-regulated in MHC-CELFΔ mice were down-regulated in MCKCUG-BP1 mice. Neither HOPX nor FHL2 completely repress SRF activity on all of its targets [12], [23]. Thus, the combination of HOPX and FHL2 down-regulation in the MHC-CELFΔ heart may have a greater stimulatory effect on SRF activity than the repressive effect of up-regulating FHL2 alone in MCKCUG-BP1 hearts. Congruent with this, *Fos* is not repressed in the MCKCUG-BP1 heart, and *Fos* expression has been reported to be unresponsive to FHL2 [12]. Strikingly, even though SRF plays a pivotal role in the maintenance of cardiac structure and function during development and pathogenesis, HOPX and FHL2 levels were not significantly affected in the two heart-specific SRSF1 and SRSF2 knockout mice [20], [21]. Taken together, the data from all of these mouse models indicate that the level of CELF activity plays a specific role in modulating the level of SRF activity in heart muscle via its interacting proteins.

Two SRF targets, *Casq1* and *Tpm2*, were up-regulated in response to either CELF repression or CELF1 over-expression. Neither of these genes is up-regulated in several other mouse models of dilated cardiomyopathy or cardiac hypertrophy [20], [21], [38]. This suggests that while these genes may respond specifically to dysregulation of CELF proteins, the modulation of SRF activity by HOPX and FHL2 is not the only determinant of their steady state levels.

The mechanism by which CELF-mediated alternative splicing regulates *Hopx* and *Fhl2* levels is currently unknown. Alternative splicing can lead to changes in transcript and/or protein levels through the introduction or removal of regulatory elements in the untranslated regions that control transcript stability, localization, or translation [39]. In addition, some transcript variants contain premature termination codons, which can target a transcript for degradation via the nonsense-mediated mRNA decay (NMD) pathway [40]. We were unable to detect any change in *Hopx* or *Fhl2* alternative splicing in MHC-CELFΔ hearts compared to wild type (data not shown), but a variant that is subject to NMD would by definition be rapidly decayed and potentially difficult to detect. It is also possible that the mechanism of *Hopx* and *Fhl2* down-regulation is indirect, through the regulation of a factor that in turn modulates *Hopx* and *Fhl2* levels. Little is currently known about the regulation of *Hopx* or *Fhl2* expression in the heart. The elucidation of additional CELF targets could help establish this link.

The extent to which HOPX and FHL2 levels are down-regulated correlates with the development of cardiomyopathy in MHC-CELFΔ severe line males (Figure 7). Surprisingly, however, HOPX and FHL2 are down-regulated to a greater extent in the mild line than the severe line (Figures 4 and 5). This may be the result of differences in integration site and/or genetic drift between the two lines. Alternatively, an additional compensatory mechanism may be activated that partially restores HOPX and FHL2 expression in the severe line, but not in the less dysfunctional mild line. In any case, the degree of de-repression of SRF alone is insufficient to explain differences in pathogenesis. Notably, the extent to which alternative splicing is disrupted also correlates with disease in the severe line males, as well as in females of the two lines ([11] and Figure S4). Thus, it is likely the combined effects of dysregulation of SRF and alternative splicing that ultimately determine the extent to which contractile function in the heart is disrupted and dilated cardiomyopathy ensues.

### Conclusions

**Figure 8:**
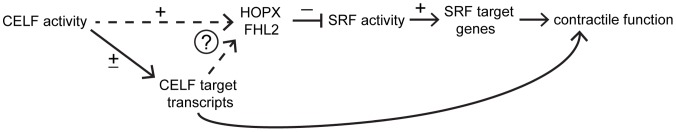
CELF-mediated alternative splicing regulates contractile function via transcriptional and post-transcriptional control mechanisms. SRF regulates the transcription of genes involved in contractile apparatus structure and function. HOPX and FHL2 bind to SRF and inhibit its ability to activate target gene transcription. We propose a model in which CELF-mediated alternative splicing regulates the SRF transcriptional program via modulation of *Hopx* and/or *Fhl2* levels. Reduced CELF-mediated alternative splicing activity in the hearts of MHC-CELFΔ mice leads to a decrease in *Hopx* and *Fhl2*, and a complementary increase in SRF target gene expression. Over-expression of CELF1 in the hearts of MCKCUG-BP1 mice leads to an increase in *Fhl2*, though not *Hopx*, and a decrease in some SRF target genes. At this time, it is not clear whether *Fhl2* and *Hopx* transcript levels are regulated directly or indirectly by CELF activity. In addition, CELF proteins regulate the alternative splicing of some cardiac transcripts known to encode proteins involved in contractile function, such as *Ank2*. Thus, the cardiac CELF-mediated alternative splicing program likely regulates contractile function both by modulating the activity of a key transcription factor that controls the expression levels of contractile genes, and by directing the production of specific variants that affect contractile performance.

Through the regulation of the SRF transcriptional network and the regulation of alternative splicing of cardiac transcripts, CELF proteins directly or indirectly exert control over cardiac gene expression at both transcriptional and post-transcriptional levels (Figure 8). CELF-mediated alternative splicing programs in the heart may therefore represent an important regulatory node for modulating cardiac function during development, health, and disease.

## Supporting Information

Figure S1 **Hierarchical clustering of raw data.** Wild type samples from both lines clustered together when hierarchical clustering was performed using correlation distances. Similar clustering was seen when using normalized and quality control-filtered data sets (data not shown).
(PDF)

Figure S2 **Gene ontology analysis identified enrichment of biological processes altered in the hearts of MHC-CELFΔ mice.** Boxes are labeled with GO ID, term definition, and P value. Red arrows represent relationships between two enriched GO terms, black solid arrows represent relationships between enriched and unenriched terms, and black dashed arrows represent relationships between two unenriched GO terms. Red boxes represent terms enriched in the mild line, green boxes represent terms enriched in the severe line, and yellow boxes represent terms enriched in both lines (p1 = P value in mild line, p2 = P value in severe line). The degree of color saturation reflects the significance of enrichment of the corresponding GO term.
(PDF)

Figure S3 **Gene ontology analysis identified enrichment of cellular components altered in the hearts of MHC-CELFΔ mice.** Boxes are labeled with GO ID, term definition, and P value. Red arrows represent relationships between two enriched GO terms, black solid arrows represent relationships between enriched and unenriched terms, and black dashed arrows represent relationships between two unenriched GO terms. Red boxes represent terms enriched in the mild line, green boxes represent terms enriched in the severe line, and yellow boxes represent terms enriched in both lines (p1 = P value in mild line, p2 = P value in severe line). The degree of color saturation reflects the significance of enrichment of the corresponding GO term.
(PDF)

Figure S4 **Alternative splicing of** ***Ank2*** **is dysregulated in the hearts of MHC-CELFΔ severe line females.** Total RNA was harvested from the hearts of wild type (WT), mild and severe (Sev) line MHC-CELFΔ females at three and 24 weeks of age. The alternative splicing of *Ank2* exon 21 was evaluated by RT-PCR. (A) Mean values+standard error of the mean are shown for three individuals per group. (B) Representative autoradiographs from the RT-PCR gels are shown. Reactions for each time point were run on the same gel; intervening replicate or blank lanes have been excised.
(PDF)

Table S1 **Real time primer sequences for the SRF pathway genes.**
(DOC)

Table S2 **Primary and secondary antibodies used for western blot analyses.**
(DOC)

Table S3 **Significantly affected genes in MHC-CELFΔ hearts.**
(XLS)

Table S4 **Comparison of genes affected in MHC-CELFΔ hearts with microarray data sets from cardiac-specific SRSF1 and SRSF2 knockout models.**
(XLS)
