# Supplementary figures and images for: Gene Expression Analyses Implicate an Alternative Splicing Program in Regulating Contractile Gene Expression and Serum Response Factor Activity in Mice
Source: PLoS One. 2013 Feb 20;8(2):e56590. doi: 10.1371/journal.pone.0056590 (PMC3577904; doi:10.1371/journal.pone.0056590)

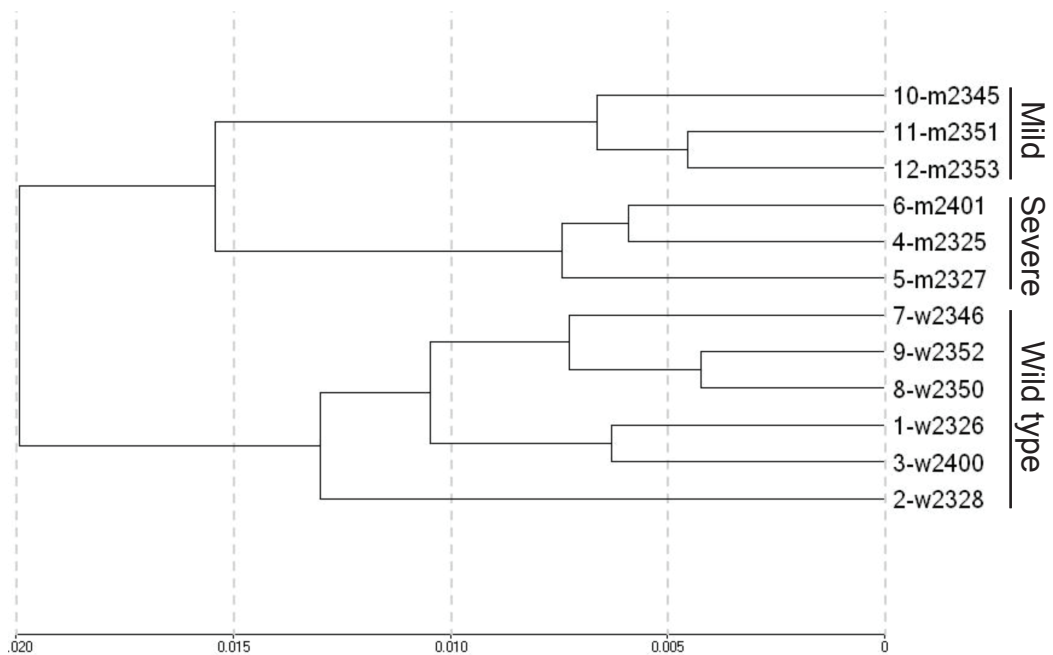

Supplement: Figure S1 — Hierarchical clustering of raw data. Wild type samples from both lines clustered together when hierarchical clustering was performed using correlation distances. Similar clustering was seen when using normalized and quality control-filtered data sets (data not shown). (PDF) [file pone.0056590.s001.pdf]

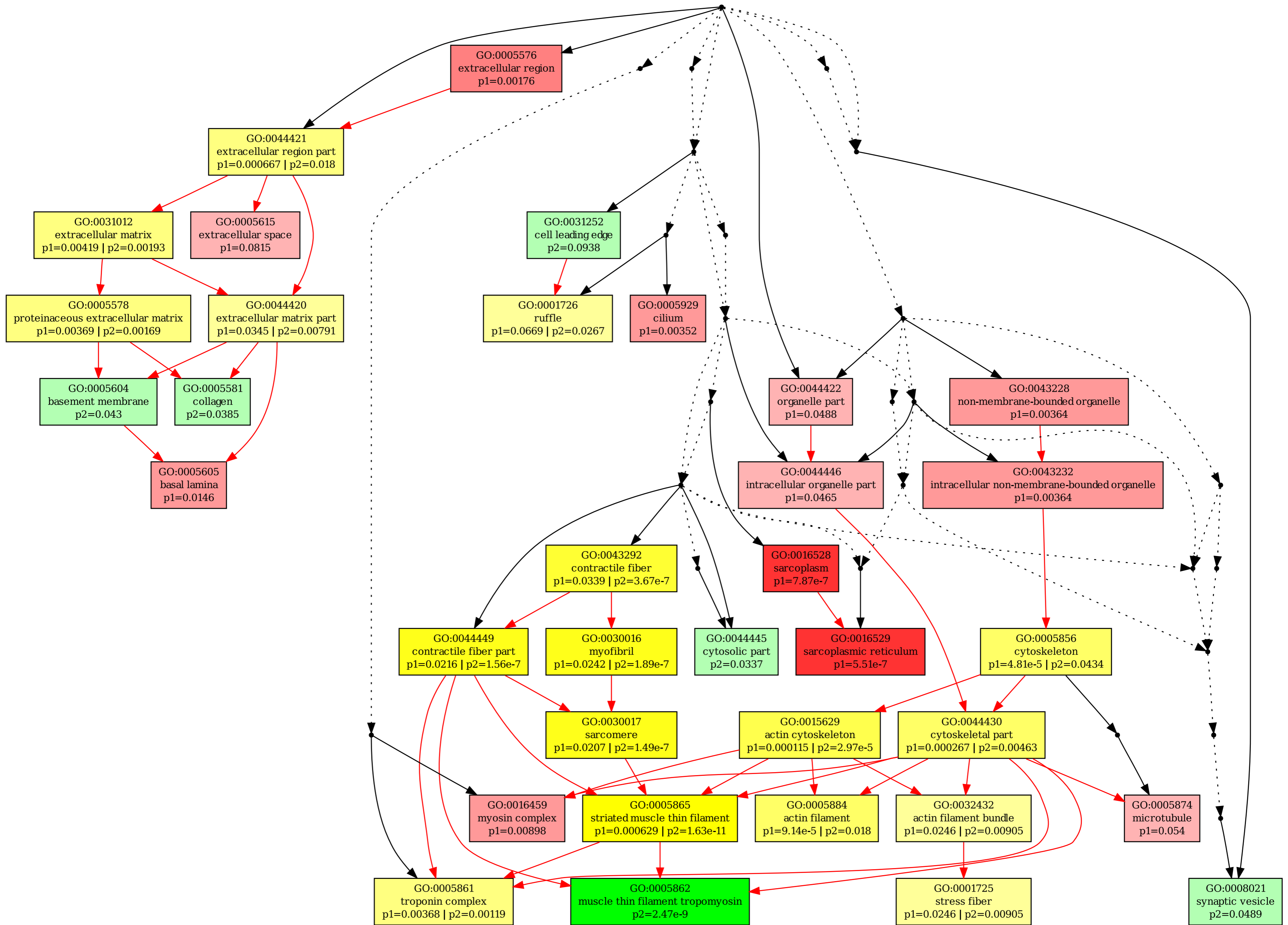

Supplement: Figure S2 — Gene ontology analysis identified enrichment of biological processes altered in the hearts of MHC-CELFΔ mice. Boxes are labeled with GO ID, term definition, and P value. Red arrows represent relationships between two enriched GO terms, black solid arrows represent relationships between enriched and unenriched terms, and black dashed arrows represent relationships between two unenriched GO terms. Red boxes represent terms enriched in the mild line, green boxes represent terms enriched in the severe line, and yellow boxes represent terms enriched in both lines (p1 = P value in mild line, p2 = P value in severe line). The degree of color saturation reflects the significance of enrichment of the corresponding GO term. (PDF) [file pone.0056590.s002.pdf]

*Ank2* exon 21

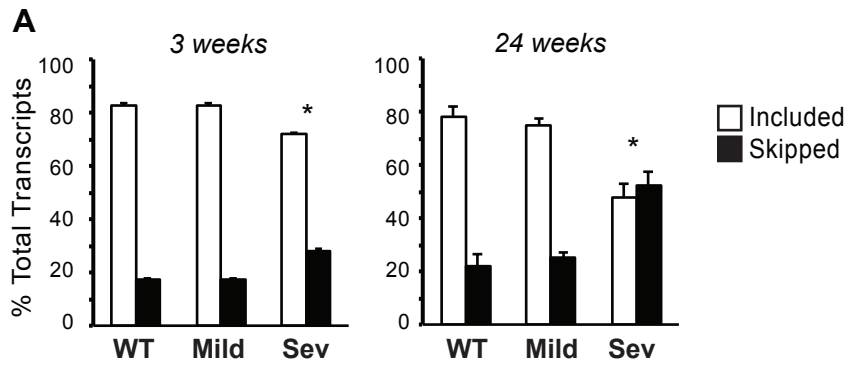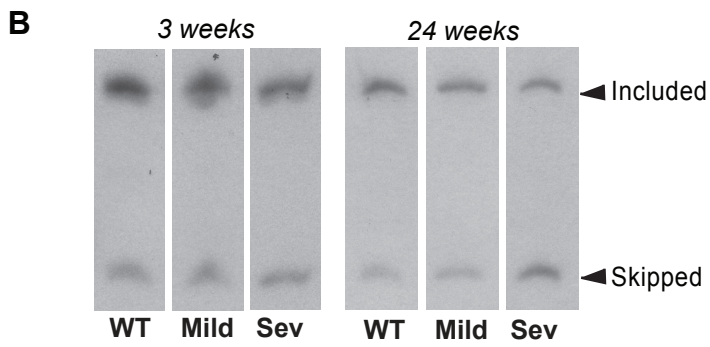

Supplement: Figure S4 — Alternative splicing of Ank2 is dysregulated in the hearts of MHC-CELFΔ severe line females. Total RNA was harvested from the hearts of wild type (WT), mild and severe (Sev) line MHC-CELFΔ females at three and 24 weeks of age. The alternative splicing of Ank2 exon 21 was evaluated by RT-PCR. (A) Mean values+standard error of the mean are shown for three individuals per group. (B) Representative autoradiographs from the RT-PCR gels are shown. Reactions for each time point were run on the same gel; intervening replicate or blank lanes have been excised. (PDF) [file pone.0056590.s004.pdf]
