# Supplementary material for: Gene Expression Analyses Implicate an Alternative Splicing Program in Regulating Contractile Gene Expression and Serum Response Factor Activity in Mice
Source: PLoS One. 2013 Feb 20;8(2):e56590. doi: 10.1371/journal.pone.0056590 (PMC3577904; doi:10.1371/journal.pone.0056590)
Supplement: Table S1 — Real time primer sequences for the SRF pathway genes. (DOC) [file pone.0056590.s005.doc]

**Table S1. Real time primer sequences for the SRF pathway genes**

| **Genes** | **Forward primer** | **Reverse primer** |
| --- | --- | --- |
| *Srf* | cacctaccaggtgtcggaat | gtctggattgtggaggtggt |
| *Fhl2* | gccaggtacccgcaagatg | gcttctcatagcagggcacg |
| *Hopx* | caacttcaacaaggtcaacaagc | gcttaaaccatttctgcgtc |
| *Acta1* | gtgaccacagctgaacgtgag | ctccagggaggaggaagag |
| *Casq1* | cagcaaggtggcaaagaagc | cacgaagctgacaatctcc |
| *Ctgf* | gtgtgcactgccaaagatg | ctgcacaggggcacgcag |
| *Nppb* | gcacaagatagaccggatcg | cttcaaaggtggtcccagag |
| *Fos* | tcctactaccattccccagc | tggcactagagacggacaga |
| *Egr1* | gagcgaacaaccctatgagc | tgggataactcgtctccacc |
| *Rcan1* | gctcagactttacacataggaag | gatcgtaatttatgacgggggtg |
| *Fhl1* | gatggcaagatcctgtgcaac | cacgttctggtctcctgc |
| *Tpm2* | gagagcgagagaggaatgaagg | cctcagcgatgtgcttgg |
| *Gapdh* | tcgtcccgtagacaaaatgg | ttgaggtcaatgaaggggtc |
