# Supplementary material for: Gene Expression Analyses Implicate an Alternative Splicing Program in Regulating Contractile Gene Expression and Serum Response Factor Activity in Mice
Source: PLoS One. 2013 Feb 20;8(2):e56590. doi: 10.1371/journal.pone.0056590 (PMC3577904; doi:10.1371/journal.pone.0056590)
Supplement: Table S2 — Primary and secondary antibodies used for western blot analyses. (DOC) [file pone.0056590.s006.doc]

**Table S2. Primary and secondary antibodies used for western blot analyses**

| Protein | Primary Antibody | Secondary Antibody |
| --- | --- | --- |
| HOPX | Rabbit polyclonal (Sigma cat. SAB4502543) | Goat anti-rabbit HRP (Calbiochem cat. 401393) |
| FHL2 | Mouse monoclonal (Santa Cruz Biotechnology cat. sc-52667) | Goat antimouse light chain specific (Jackson Immunoresearch cat. 115-035-174) |
| SRF | Rabbit polyclonal (Santa Cruz Biotechnology cat. sc:335) | Goat anti-rabbit HRP (Calbiochem cat. 401393) |
| FHL1 | Mouse monoclonal (Sigma cat. WH0002273M1) | Goat antimouse light chain specific (Jackson Immunoresearch cat. 115-035-174) |
| CASQ1 | Rabbit affinity isolated (Sigma cat. C0618) | Goat anti-rabbit HRP (Calbiochem cat. 401393) |
| ACTA1 | Rabbit polyclonal (Sigma cat. SAB4502543) | Goat anti-rabbit HRP (Calbiochem cat. 401393) |
| CELF1 | Mouse monoclonal (Santa Cruz Biotechnology cat. sc-20003) | Goat antimouse light chain specific (Jackson Immunoresearch cat. 115-035-174) |
| GAPDH | Mouse monoclonal (Biogenesis cat. 4699-9555) | Goat anti-mouse-HRP (Calbiochem cat. DC02L) |
