# Supplementary material for: Gene Expression Analyses Implicate an Alternative Splicing Program in Regulating Contractile Gene Expression and Serum Response Factor Activity in Mice
Source: PLoS One. 2013 Feb 20;8(2):e56590. doi: 10.1371/journal.pone.0056590 (PMC3577904; doi:10.1371/journal.pone.0056590)

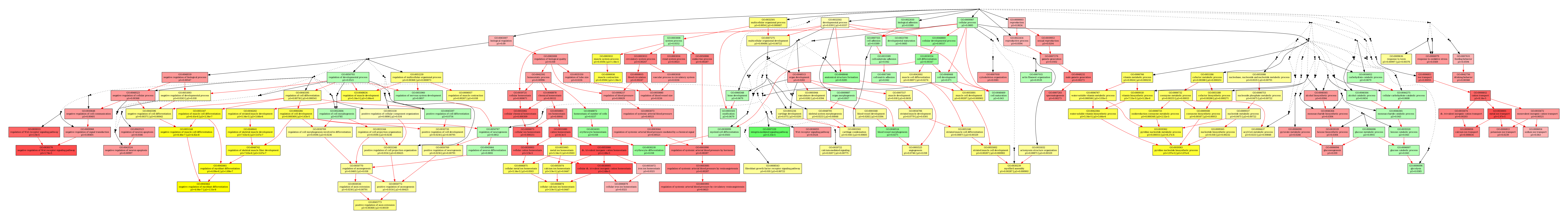

Supplement: Figure S3 — Gene ontology analysis identified enrichment of cellular components altered in the hearts of MHC-CELFΔ mice. Boxes are labeled with GO ID, term definition, and P value. Red arrows represent relationships between two enriched GO terms, black solid arrows represent relationships between enriched and unenriched terms, and black dashed arrows represent relationships between two unenriched GO terms. Red boxes represent terms enriched in the mild line, green boxes represent terms enriched in the severe line, and yellow boxes represent terms enriched in both lines (p1 = P value in mild line, p2 = P value in severe line). The degree of color saturation reflects the significance of enrichment of the corresponding GO term. (PDF) [file pone.0056590.s003.pdf]
